# Pharmacogenetic Variation in Neanderthals and Denisovans and Implications for Human Health and Response to Medications

**DOI:** 10.1093/gbe/evad222

**Published:** 2023-12-05

**Authors:** Tadeusz H Wroblewski, Kelsey E Witt, Seung-been Lee, Ripan S Malhi, David Peede, Emilia Huerta-Sánchez, Fernando A Villanea, Katrina G Claw

**Affiliations:** Department of Biomedical Informatics, Colorado Center for Personalized Medicine, University of Colorado Anschutz Medical Campus, Aurora, Colorado, USA; Center for Human Genetics and Department of Genetics and Biochemistry, Clemson University, South Carolina, USA; Precision Medicine Institute, Macrogen Inc., Seoul, Republic of Korea; Department of Anthropology and Carl R. Woese Institute for Genomic Biology, University of Illinois Urbana-Champaign, Illinois, USA; Department of Ecology, Evolution, and Organismal Biology and Center for Computational and Molecular Biology, Brown University, Providence, Rhode Island, USA; Institute at Brown for Environment and Society, Brown University, Providence, Rhode Island, USA; Department of Ecology, Evolution, and Organismal Biology and Center for Computational and Molecular Biology, Brown University, Providence, Rhode Island, USA; Department of Anthropology, University of Colorado Boulder, Colorado, USA; Department of Biomedical Informatics, Colorado Center for Personalized Medicine, University of Colorado Anschutz Medical Campus, Aurora, Colorado, USA

**Keywords:** pharmacogenetics, archaic, genetics, ancient DNA

## Abstract

Modern humans carry both Neanderthal and Denisovan (archaic) genome elements that are part of the human gene pool and affect the life and health of living individuals. The impact of archaic DNA may be particularly evident in pharmacogenes—genes responsible for the processing of exogenous substances such as food, pollutants, and medications—as these can relate to changing environmental effects, and beneficial variants may have been retained as modern humans encountered new environments. However, the health implications and contribution of archaic ancestry in pharmacogenes of modern humans remain understudied. Here, we explore 11 key cytochrome P450 genes (*CYP450*) involved in 75% of all drug metabolizing reactions in three Neanderthal and one Denisovan individuals and examine archaic introgression in modern human populations. We infer the metabolizing efficiency of these 11 *CYP450* genes in archaic individuals and find important predicted phenotypic differences relative to modern human variants. We identify several single nucleotide variants shared between archaic and modern humans in each gene, including some potentially function-altering mutations in archaic *CYP450* genes, which may result in altered metabolism in living people carrying these variants. We also identified several variants in the archaic *CYP450* genes that are novel and unique to archaic humans as well as one gene, *CYP2B6*, that shows evidence for a gene duplication found only in Neanderthals and modern Africans. Finally, we highlight *CYP2A6*, *CYP2C9*, and *CYP2J2*, genes which show evidence for archaic introgression into modern humans and posit evolutionary hypotheses that explain their allele frequencies in modern populations.

**Significance**

Pharmacogenes code for enzymes that process exogenous compounds to neutralize or repurpose them, and the most important of these enzymes are expressed by the cytochrome P450 (or *CYP450*) genes. Here, we characterize variation in 11 *CYP450* genes in archaic human (Neanderthal and Denisovan) genomes and compare them to the variants we observe in modern humans. We find that archaic humans carry similar *CYP450* variants to modern humans—some show evidence for being introduced to modern humans through gene flow with archaic humans, while others are unique to archaic humans and would likely have negatively impacted enzyme function, suggesting that selection may have shaped the archaic *CYP450* variants that are present in humans today.

## Introduction

The cytochrome P450 (*CYP450*) genes encode oxidase enzymes that function in metabolism of endogenous small molecules and in detoxification of exogenous (or xenobiotic) compounds. This gene family is present in all mammals, with 57 active genes and 58 pseudogenes coding for CYP450 enzymes in humans (Thomas 2007). A subset of these *CYP450* genes code for enzymes responsible for metabolizing xenobiotic substrates, and these have been studied extensively because of their roles in the absorption, distribution, metabolism, and excretion (ADME) of pharmaceuticals and their associated significance for drug development. The evolution of xenobiotic CYP450 enzymes may be driven by organisms’ need to metabolically detoxify foreign compounds—often toxic chemicals produced by plants, fungi, and bacteria—in the local environment. Because of their direct role in mediating environmental challenges, the xenobiotic-metabolizing *CYP450* genes present high allele diversity in human populations and often show evidence of directional selection as a response to unstable environmental interactions, such as toxin and pathogen exposure (Thomas 2007; Fuselli et al. 2010). The *CYP450* gene family is known to evolve very quickly via gene duplications, which increase metabolizer function, or by loss-of-function mutations, which decrease metabolizer function (Thomas 2007).

It has also been suggested that the shift from hunting and gathering to food production in humans may have profoundly changed the selective effect of certain CYP450 enzymes (Fuselli et al. 2010; Fuselli 2019). Although these genes have been studied extensively in humans and other mammals, the genetic variation in archaic individual pharmacogenes and its relation to modern human pharmacogene variation has not been addressed. Genetic variation in ADME genes varies extensively across modern human populations and identifying alleles inherited through archaic introgression may be informative about the origin of specific pharmacogenetic (i.e., genes that modulate the body's response to pharmaceuticals; PGx) variants and their phenotypes, including metabolizer status.

Throughout human evolutionary history, our species has adapted to numerous distinct and varied challenging environments, which present the need to metabolize new xenobiotic substances, such as food, pollutants, and, most recently, medications (Fan et al. 2016). Modern humans encountered new environmental challenges as they dispersed first throughout and then outside of the African continent, but they also encountered other hominin species already adapted to those environments (i.e., Neanderthals and Denisovans) (Durvasula and Sankararaman 2020; Bergström et al. 2021). The direct sequencing of multiple Neanderthal and Denisovan genomes has revealed a complex history of admixture between these archaic humans and the ancestors of modern humans (Browning et al. 2018; Villanea and Schraiber 2019). Most modern humans carry a small but significant portion of archaic ancestry, which has been targeted by natural selection (Sankararaman et al. 2016). Purifying selection—or negative natural selection—has removed many archaic variants in functional genomic regions (Petr et al. 2019; Zhang et al. 2020; Schaefer et al. 2021), and some archaic variants may have been lost through genetic bottlenecks or drift in modern human populations. Despite strong purifying selection, there are still functional regions for which archaic variants are found at very high frequency in living humans through the effects of positive natural selection (Mendez et al. 2012; Huerta-Sánchez et al. 2014; Racimo et al. 2015; Dannemann et al. 2016). One of the most well-known examples of adaptation through archaic admixture is *EPAS1*, a gene that is involved in the response to hypoxia. Tibetans have a variant of *EPAS1* at extremely high frequency that has conferred resistance to hypoxic stress in high-altitude environments and was acquired through admixture with Denisovans (Huerta-Sánchez et al. 2014; Zhang et al. 2021).

The *CYP450* genes offer a promising target for adaptation through archaic introgression as humans expanded their geographic range and encountered novel exogenous substances. Evidence suggests that *CYP450* variants have evolved in response to dietary or environmental toxins (Fuselli et al. 2010; Fan et al. 2016; Fuselli 2019). Neanderthals and Denisovans may have possessed *CYP450* variants fine-tuned to metabolizing substances found in their native habitats of Eurasia and Siberia. As modern humans moved outside of Africa, they likely faced novel environmental factors which may have influenced selective pressures; therefore, advantageous archaic *CYP450* variants introduced to modern humans through archaic admixture may have been retained in the modern human gene pool by natural selection. Previous work has shown that some pharmacogenes have been targeted for selection in populations outside of Africa, including variants of *CYP3A4* and *CYP3A5* which impact salt metabolism (Thompson et al. 2004), supporting our hypothesis that pharmacogenes may have been subject to evolutionary pressures as humans moved into new environments. Recent research has also suggested that some pharmacogene haplotypes (CYP2C8*3 and CYP2C9*2) show evidence for being introgressed from Neanderthals (Haeggström et al. 2022).

Here, we examine genetic variation and predict metabolizer phenotypes in 11 *CYP450* genes in three Neanderthal and one Denisovan individuals using publicly available high-resolution whole genome sequencing data. Out of the 57 functional human *CYP450* genes, these 11 genes are responsible for up to 75% of the metabolism of commonly prescribed drugs and other xenobiotic substrates (Ekins and Wrighton 1999; Evans and Relling 1999; Chen et al. 2019; Esteves et al. 2021), which could have direct implications for modern human health, disease, and drug safety and efficacy. In addition, we examine the patterns of allele sharing between archaic and modern humans to identify haplotypes that may have been introgressed. This study aims to increase our understanding of introgressed genetic variants and the different selective regimes and environments acting on these enzymes.

## Results

### CYP450 Variation in Neanderthals and Denisovans

**Fig. 1.**
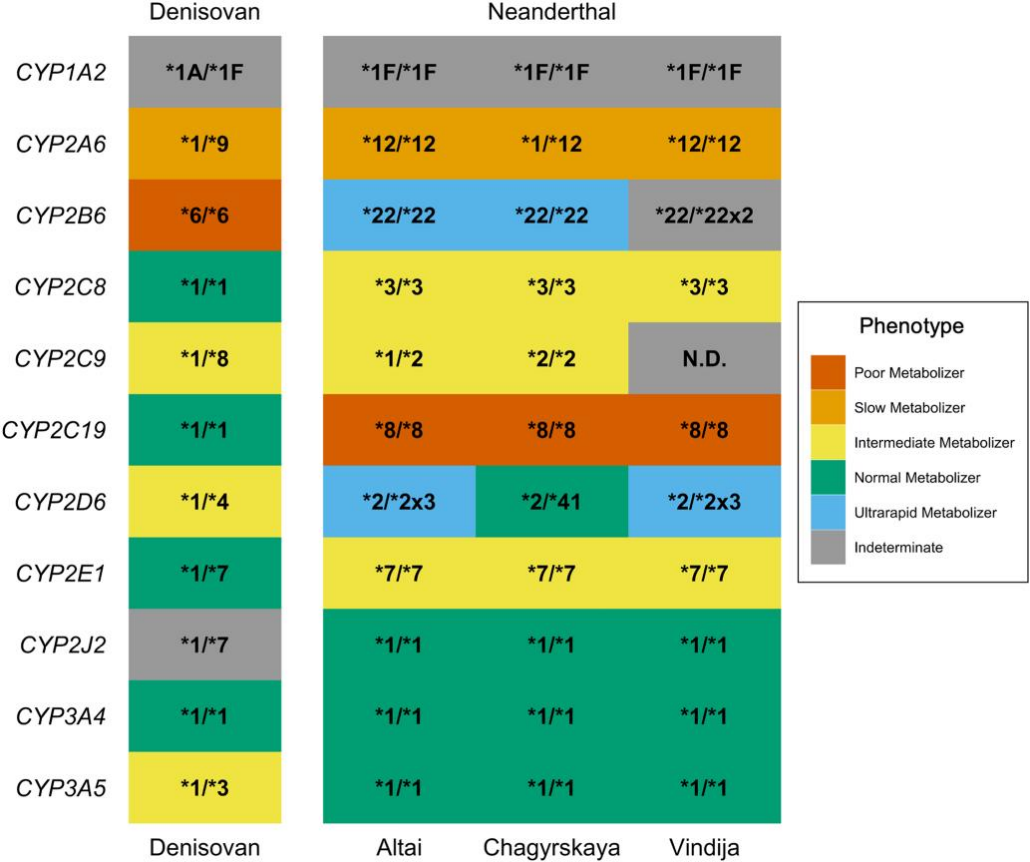
Phased diplotypes and predicted metabolizer phenotypes identified in archaic individuals. Heatmap showing the primary star allele diplotypes for each gene from the three Neanderthal and one Denisovan individuals. Each diplotype was determined as the two phased haplotypes, which are detected with the variant and read data from each gene. The fill of each tile represents the predicted phenotype determined by the combined activity score associated with the presented diplotype as identified in modern humans. Indeterminate phenotypes indicate that the functional effect of the diplotype has not been determined. A diplotype was considered nondeterminant (N.D.) if there was a lack of read coverage at specific variant locations. Frequencies from different human populations in the 1000 Genomes Project are reported in supplementary table S8, Supplementary Material online.

In this study, we investigated genetic variation in the eleven most important *CYP450* genes for xenobiotic metabolism: *CYP1A2, CYP2A6, CYP2B6, CYP2C8, CYP2C9, CYP2C19, CYP2D6, CYP2E1, CYP2J2, CYP3A4,* and *CYP3A5*. We started by examining the pharmacogene haplotypes present in the archaic Neanderthal and Denisovan individuals. Pharmacogene haplotypes are typically referred to as star alleles (Gaedigk et al. 2018), which are typically associated with protein activity levels. We used variant and depth of coverage data to identify the archaic star alleles using previously validated methods (Lee, Wheeler, Patterson, et al. 2019; Lee, Wheeler, Thummel, et al. 2019). The phased diplotype for each archaic individual was identified as the two primary haplotype calls based on the presence or absence of star allele-defining nucleotides and structural variants (fig. 1, supplementary table S4, Supplementary Material online).

Pharmacogene diplotypes were identified for all eleven of the pharmacogenes studied for all four archaic individuals, with the exception of the Vindija diplotype for *CYP2C9*, which was indeterminate due to very low read coverage at informative variant sites (fig. 1). For each diplotype, the predicted metabolizer status phenotype was assessed as designated by the Pharmacogene Variation Consortium (https://www.pharmvar.org/) (Gaedigk et al. 2018). We observed variability in the metabolic rate of the archaic pharmacogenes, ranging from “ultra-rapid” (i.e., the enzyme would break down compounds much faster than expected) to “poor” (i.e., the enzyme is virtually nonfunctional) metabolizers (fig. 1). One nuance of this analysis is that the impact of being a slower or faster metabolizer is not necessarily universally positive or negative but is context-dependent based on multiple factors, including whether breaking down a compound activates or inactivates its function, as well as environmental influences and the genetic background of the individual bearing the diplotype. Additionally, the metabolizer phenotypes were established in modern humans although they are known to be important in all species, and so the phenotypes may slightly differ for the archaic individuals. Additionally, any single nucleotide variants (SNVs) unique to archaic humans would not have been identified or assessed for their functional impact on the metabolizer phenotype. 1KG frequencies for diplotypes reported in figure 1 are reported in supplementary table S8, Supplementary Material online.

### Pharmacogene Structural Variation in Archaic Humans

**Fig. 2.**
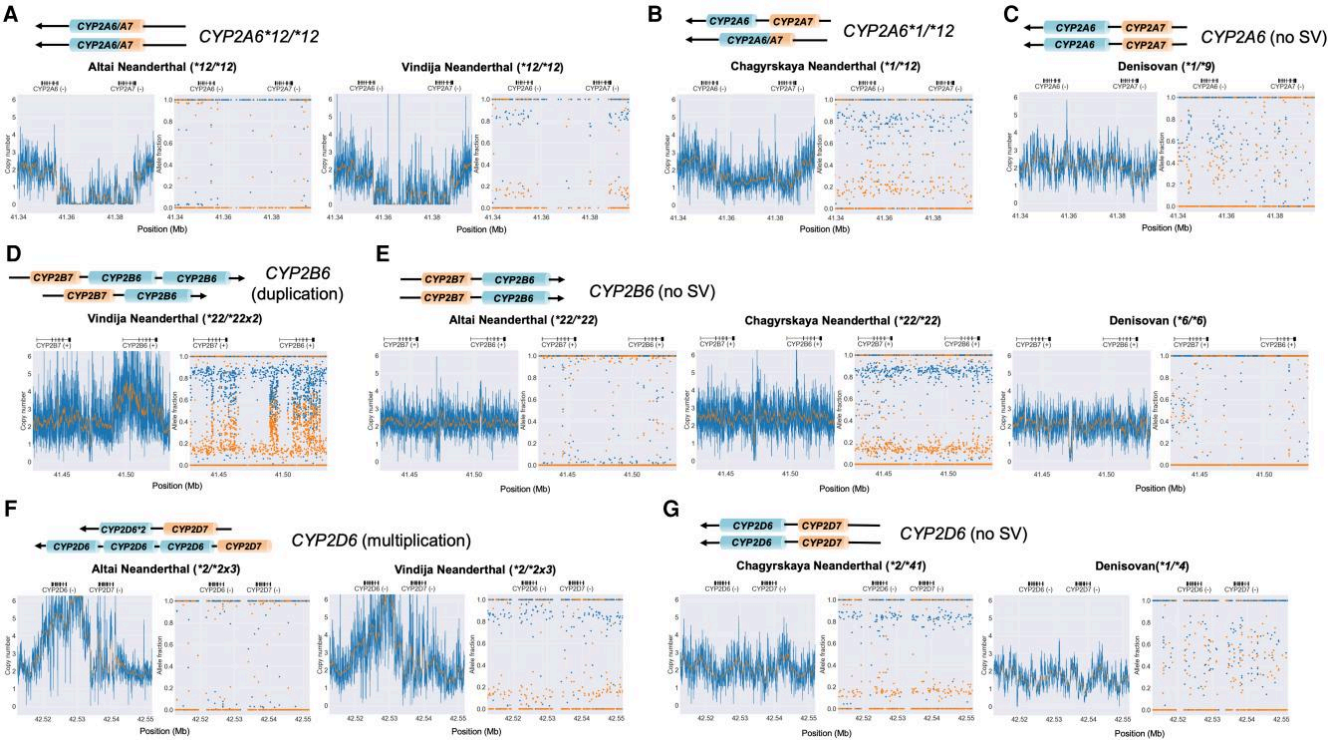
Structural variants detected in CYP2A6, CYP2D6, and CYP2B6. Copy number variation plots (left) demonstrating the structural variation and allele fraction plots (right) showing allelic fraction for each Neanderthal and Denisovan individual across CYP2A6, CYP2D6, and CYP2B6. The CYP2A6/A7 structural variant, *12, identified for the (*A*) Altai Neanderthal and Vindija Neanderthal which both present a *12/*12 diplotype, characterized by a partial deletion hybridization event in both gene copies. (*B*) The Chagyrskaya Neanderthal demonstrated a *1/*12 diplotype and (*C*) the Denisovan individual presented no structural variation. For CYP2B6, (*D*) the Vindija Neanderthal displayed a gene duplication event, while (*E*) no structural variation was identified for the Altai Neanderthal, Chagyrskaya Neanderthal, and Denisovan individual. In CYP2D6, (*F*) the Altai and Vindija Neanderthals presented a gene multiplication while the (*G*) Chagyrskaya Neanderthal and Denisovan individual presented no structural variation in CYP2D6. Copy number plots are estimated based on read depth data (displayed as the copy number normalized to the VDR control gene region) with the center line indicating the copy number assessment for each gene. Allele fraction plots display the allele frequency of each variant for the two haplotypes, which demonstrates the allelic decomposition after entifying structural variation. For each structural variant, there is a schematic representation of the predicted structure of each gene, with an arrow indicating the direction of transcription. The hg19 genetic coordinates are presented on the *x* axis with the gene regions indicated directly above.

Structural variation (SV) was identified in three of the 11 pharmacogenes investigated—*CYP2A6, CYP2B6,* and *CYP2D6* (fig. 2). The *CYP2A6* gene is expressed mainly in the liver and represents between 1% and 10% of the total liver CYP450 protein (Haberl et al. 2005). The CYP2A6 enzyme metabolizes drugs and pro-carcinogenic compounds including tegafur (Komatsu et al. 2000), valproic acid (Kiang et al. 2006; Tan et al. 2010), and coumarin (Miles et al. 1990) and is the primary enzyme involved in nicotine metabolism (Hukkanen et al. 2005). The Altai and Vindija Neanderthals presented the homozygous partial deletion hybrid CYP2A6*12 variant, while the Chagyrskaya Neanderthal was heterozygous for the *12 haplotype (fig. 2*A*–*C*). This allele has been identified as a hybrid between *CYP2A6* and the inactive gene *CYP2A7,* with exons 1 and 2 originating from *CYP2A7*, and exons 3–9 originating from *CYP2A6*. This allele causes a 50% reduction in CYP2A6 protein levels and a 40% decrease in CYP2A6 coumarin 7-hydroxylation activity in modern humans and has been found at low frequencies in some global sampled populations (Oscarson et al. 2002; Hukkanen et al. 2005). At present, more than 10 different allelic variants are known that cause absent or reduced enzyme activity, including multiple haplotypes created by unequal crossover and gene conversion reactions between *CYP2A6* and *CYP2A7,* suggesting that eliminating or reducing *CYP2A6* function is a recurring evolutionary strategy (Oscarson et al. 2002; Benowitz et al. 2006). It has been proposed that the slow metabolizing of some plant secondary metabolites may be adaptive as high levels of these toxins in human tissues may act as a deterrent to parasites (Sullivan et al. 2008; Hagen et al. 2009, 2013), perhaps explaining why this hybrid allele may have evolved multiple times.

**Fig. 3.**
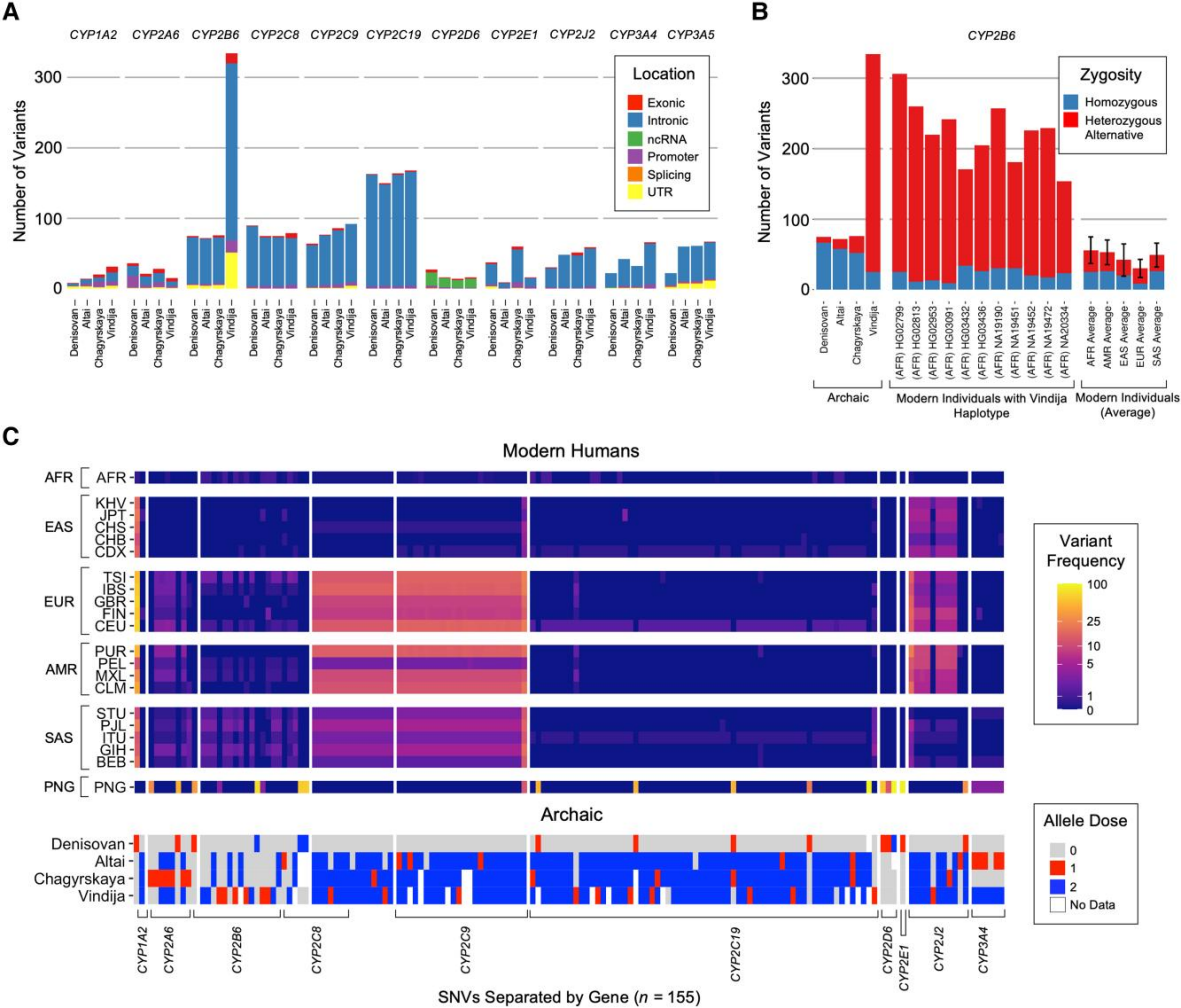
CYP450 genetic variation in archaic individuals and shared with modern humans. (*A*) The total number of SNVs identified in each archaic individual displayed as a stacked bar plot. Each color displays the relative variant location identified using ANNOVAR with 3′ and 5′ untranslated regions grouped together (UTR), and the promoter region (Promoter) characterized as any upstream variants that are within 2000bp of the gene. (*B*) A larger number of variants were identified in *CYP2B6* in the Vindija Neanderthal. Bar plots have been faceted by the archaic individuals, the modern individuals identified to share the Vindija *CYP2B6* haplotype, and the average number of variants for 10 modern individuals from each superpopulation of the 1KG Project. Variants are colored by zygosity (i.e., heterozygous if the variant site has one alternative allele and homozygous alternative if the variant site has two alternative alleles at that position). The height of each stacked bar plot represents the total number of variants for each individual and the number of variants for the modern individuals have been reported as the mean with error bars representing the standard deviation. (*C*) A subset of archaic variants (*n* = 155) was identified to have an allele frequency <1% in 1KG-AFR-like populations and >1% in at least one non-1KG-AFR-like population. Subpopulations have been grouped as African-like (1KG-AFR-like), East Asian (1KG-EAS-like), European (1KG-EUR-like), admixed American (1KG-AMR-like), South Asian (1KG-SAS-like), and Papuan (SGD-PNG-like) and allele frequencies for each population are shown on the gradient heatmap. The allele dose of each archaic variant is shown in the heatmap on the bottom, with an allele dose of 0 representing homozygous reference and an allele dose of 2 representing homozygous alternative.

We identified a gene duplication event in *CYP2B6* in the Vindija Neanderthal, with a *22/*22 × 2 diplotype (fig. 2*D* and *E*). CYP2B6 is responsible for metabolizing drugs including the prodrug cyclophosphamide (Huang et al. 2000; Zanger and Klein 2013); efavirenz, a non-nucleoside reverse transcriptase inhibitor (Ward et al. 2003; Desta et al. 2007; Zanger and Klein 2013); the antidepressant, bupropion (Faucette et al. 2000; Hesse et al. 2000; Zanger and Klein 2013); and ketamine (Desta et al. 2012). Genetic polymorphisms in *CYP2B6* have been identified to alter enzyme activity (Ariyoshi et al. 2001; Lang et al. 2001; Kirchheiner et al. 2003; Ward et al. 2003; Hofmann et al. 2008), and there have been over 37 *CYP2B6* haplotypes identified (Gaedigk et al. 2018; Desta et al. 2021). Eleven modern 1000 Genomes Project (1KG) African (AFR)-like individuals also appear to have this gene duplication (supplementary fig. S4, Supplementary Material online). Interestingly, the *CYP2B6* haplotypes in the Vindija Neanderthal and the 11 1KG-AFR-like individuals also have an elevated number of SNVs relative to other modern and archaic human pharmacogene haplotypes (fig. 3*A* and *B*). These SNVs would not have been identified by the haplotype caller if they had no phenotypic effect, or if they have not been previously validated in modern humans. The Vindija Neanderthal had 334 variant sites in *CYP2B6* with approximately 92% of these variant sites being heterozygous (fig. 3*B*), while the modern 1KG-AFR-like individuals with this haplotype had approximately 200 variant sites, with the majority heterozygous. The other archaic humans and individuals sampled from the 1KG have fewer than 100 variant sites at this gene. Given that *CYP2B6* shares significant homology with the pseudogene *CYP2B7,* which is located nearby (40.6 kb) (Zanger and Klein 2013), we assessed the read depth at the *CYP2B6* locus relative to a control gene in the Vindija Neanderthal genome and the 11 1KG-AFR-like genomes and found that the read depth between the two genes are comparable, suggesting that read mis-mapping with the paralog gene is unlikely to be the cause of the elevated number of variants in these individuals (supplementary fig. S1*A*, Supplementary Material online). The allele balance at this locus is approximately 0.33–0.67 in the Vindija Neanderthal, providing further evidence that this haplotype represents a duplication rather than mis-mapping of paralogs. The *CYP2B6* locus in the Vindija Neanderthal was also identified as highly divergent from other Neanderthals relative to the rest of the genome and is within the top 1% of ∼30-kb windows based on divergence between the Chagyrskaya and Vindija Neanderthals (pairwise distance = 0.00175, supplementary fig. S2, Supplementary Material online). Interestingly, the 1KG-AFR-like and Vindija divergent haplotypes share many SNVs but also each has many variants that are exclusive to their individual haplotypes (supplementary fig. S3, Supplementary Material online).

We also identified a gene duplication event for *CYP2D6* in Altai and Vindija Neanderthals, presenting a *2/*2 × 3 diplotype (fig. 2*F* and *G*), which is considered an ultra-rapid metabolizer. *CYP2D6* is one of the most variable of all *CYP450* genes and is subject to structural changes and copy number duplications that result in increased metabolic efficiency. Ultra-rapid alleles like CYP2D6*2 × 3 are found in 1KG-AFR-like populations (10.3%) (Zhou et al. 2017). However, most modern humans have slow-metabolizing variants, which are at higher frequencies in regions outside of Africa, and especially in the East Asia region (70.3%). This variability in *CYP2D6* phenotypes suggests that environmental factors may play a role in selection for different metabolic rates of this enzyme. Because of mapping difficulties, we were unable to determine structural variants from the chimpanzee outgroup.

### Single Nucleotide Variants in Archaic and Modern Humans

We also examined SNVs in archaic humans and compared them to the SNVs observed in modern human individuals from the 1KG (1000 Genomes Project Consortium 2015) with the addition of Papuan (PNG) individuals from the Simons Genome Diversity (SGD) Project data (Mallick et al. 2016). We identified a total of 1,623 SNVs across the eleven *CYP450* genes investigated in one Denisovan (from Denisova Cave) and three Neanderthals (from the Vindija, Denisova Cave/Altai region, and Chagyrskaya sites). Of the variants identified in the four archaic individuals, the majority were intronic or noncoding, while 6.8% (*n* = 111) were in promoter regions and 4.7% (*n* = 77) were exonic (fig. 3*A*, supplementary table S1, Supplementary Material online). We classified an SNV as archaic if it was shared between modern and archaic humans and found at a frequency of less than 1% in 1KG-AFR-like populations. We identified a total of 155 archaic SNVs that fit these criteria, including 41 found in human populations at a frequency of 10% or higher, and four that were exonic (fig. 3*C*, full list of variants in supplementary table S3, Supplementary Material online). The majority of these 155 SNVs are derived (91.0%) and of the 14 ancestral SNVs, 7 SNVs (4.5%) were also found in chimpanzees, five of which were homozygous in all chimpanzees sampled (supplementary table S3, Supplementary Material online).

In some cases, archaic and modern humans had the same star allele haplotype calls but the SNVs within these haplotypes were not shared between archaic and modern humans. For example, the CYP2A6*9 allele found in the Denisovan is also the most common allele in modern 1KG humans from South Asia and East Asia regions, which both likely overlap the geographic range of Denisovans, yet the modern human CYP2A6*9 haplotype does not contain Denisovan-specific SNVs. We also observe cases where archaic and modern humans have similar phenotypes but different genotypes. For example, all three Neanderthals have the haplotype CYP2C19*8, a slow-metabolizing variant that is present in only 0.1% of 1KG-South Asian (SAS)-like and 0.3% of 1KG-European (EUR)-like individuals (Zhou et al. 2017). While the most common *CYP2C19* haplotype, *17, confers increased transcription, another slow metabolizer haplotype (CYP2C19*2) is found at 31% in East Asia and 36% in South Asia (Zhou et al. 2017). It has been suggested that CYP2C19*2 was subject to positive selection worldwide, possibly in response to exposure to a novel toxin (Janha et al. 2014). Neanderthals may have encountered a similar environment that caused selection for a slowed CYP2C19 metabolism as well.

### Functional Significance of Archaic Pharmacogene SNVs

**Fig. 4.**
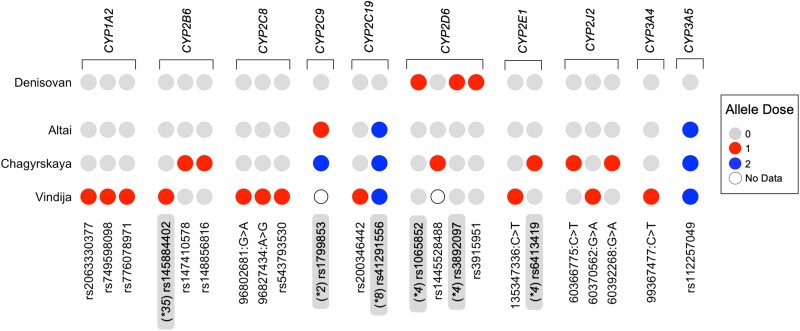
Function-altering variants in archaic individuals. Circle plot displaying the occurrence of potentially function-altering variants for each individual in the selected CYP450 genes. Variants are identified with rsID, if no rsID number was identified, variants are considered potentially novel and the hg19 position is displayed along with mutation observed (position: reference allele > alternate allele). Variants that compose known star alleles are highlighted in gray and denoted with the respective star allele preceding the rsID. Allele dosage is categorized as the number of alternate alleles at that position. Unfilled circles indicate that no call was made at the given position for that individual. An allele dose of 1 indicates an SNV is heterozygous for the reference and alternate allele, and an allele dose of 2 indicates an SNV is homozygous for the alternate allele. No variants were identified for *CYP2A6*.

We also wanted to identify SNVs that may have impacted pharmacogene function in archaic humans. Haplotype calling methods focus only on known star alleles, and so any novel SNVs found within archaic human genomes would not have been identified. Potentially function-altering SNVs were characterized using three programs that can predict functional impact of genetic variation: Combined Annotation Dependent Depletion (CADD v1.6), Sorting Intolerant From Tolerant (SIFT 4G v2.0.0), and Polymorphism Phenotyping (PolyPhen v2) (Adzhubei et al. 2010; Vaser et al. 2016; Rentzsch et al. 2019). While “potentially damaging” is the standard terminology for SNVs that alter enzyme function, in this instance, these SNVs will be referred to as “potentially function-altering” as the impact of altered pharmacogenetic enzymes is dependent both on the substrate and on the individual's genetic background. We identified 23 potentially function-altering variants in the eleven *CYP450* genes investigated (fig. 4), including 7 potentially novel variants that were not observed in any variant annotation databases utilized (see Materials and Methods).

Of the potentially function-altering variants identified, six SNVs were diagnostic for star alleles (figs. 1 and 4, supplementary table S5, Supplementary Material online). *CYP2C9* rs1799853 is the core SNV for the *2 allele and was identified in the Altai and Chagyrskaya Neanderthal individuals and is predicted to cause an intermediate metabolizer phenotype (or a slower metabolic rate) in modern humans. This SNV is also found globally in modern humans at less than 5% frequency, although 1KG-EUR-like and 1KG-AMR-like populations have higher frequencies of this allele, from 8% to 15% (supplementary table S3, Supplementary Material online). In *CYP2C19*, the primary SNV defining the *8 allele (rs41291556) was identified in the three Neanderthal individuals and confers a poor metabolizer status in modern humans (fig. 4, supplementary table S5, Supplementary Material online), although it is found at very low frequencies (0.5–1.5%) in a small number of modern human populations (supplementary table S3, Supplementary Material online). Finally, the two variants that compose the CYP2D6*4 haplotype (rs3892097 and rs1065852) were identified as heterozygotes in the Denisovan individual, which are responsible for a splicing defect and inactivation of the *CYP2D6* gene product. Combined with a normal metabolizer *1 haplotype, the Denisovan individual is predicted to have an intermediate metabolizer phenotype (fig. 4). Additionally, an exonic missense variant in *CYP2D6* was identified as heterozygous in a single SGD-Papuan-like individual (rs3915951) and was scored as likely to alter enzyme function (fig. 4, supplementary table S3, Supplementary Material online). Aside from the potentially function-altering variants identified, the majority of archaic variants present in non-1KG-AFR-like modern humans were either intronic (*n* = 140) or exonic synonymous (*n* = 4) mutations, making their impact on metabolism unclear (fig. 3*C*, supplementary table S3, Supplementary Material online).

### Introgression of Archaic CYP450 Alleles into Modern Humans

**Fig. 5.**
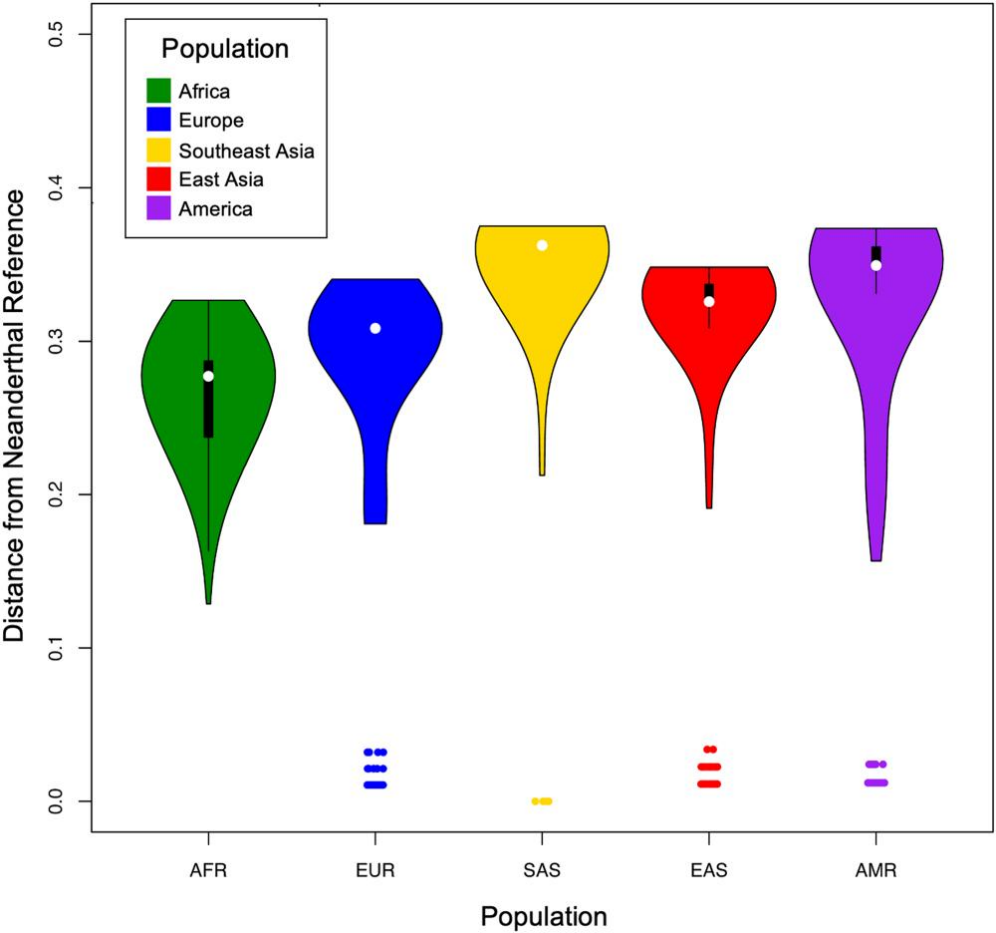
Haplostrips violin plot of CYP2J2. Haplotype distances at *CYP2J2* (hg19 1:60355979-60395470) in modern human populations relative to a Neanderthal reference haplotype (Vindija). The kernel density on each violin plot represents the abundance of individual haplotypes ordered by distance to the reference. Most human-like haplotypes sit at the same distance (furthest from the archaic reference), giving the violin plots a “flat top”. Outlier data points near lower distances represent modern 1KG human haplotypes carrying the Neanderthal-like *CYP2J2* in all populations except 1KG-AFR-like populations.

The SNVs shared between non-1KG-AFR-like populations and archaic humans may reflect patterns of past interbreeding with diverse archaic populations and genetic drift (Sankararaman et al. 2014, 2016), although other demographic scenarios such as parallel evolution or shared ancestral variation could also explain this observed pattern. To confirm if the archaic SNVs identified in modern human populations for the *CYP450* genes corresponded to an archaic haplotype inherited from archaic humans, we determine whether any of the pharmacogenes had been previously identified as part of published introgressed tracts in the individuals with those SNVs (Skov et al. 2018). In five of the pharmacogenes (*CYP2C8, CYP2C9, CYP2C19, CYP2J2,* and *CYP3A4*), over 90% of the individuals with an archaic SNV was also identified as having archaic ancestry in the region surrounding the gene (supplementary table S3, Supplementary Material online). We also calculated sequence divergence between Neanderthal and modern 1KG individuals’ *CYP450* haplotypes using Haplostrips, which calculates Manhattan distances, or the number of SNVs with different alleles between the two sequences (Marnetto and Huerta-Sánchez 2017). Of the 11 *CYP450* genes studied here, the gene with the clearest evidence for haplotype sharing between Neanderthals and modern 1KG humans was *CYP2J2*, where a Neanderthal-like haplotype was present in all non-1KG-AFR-like populations at frequencies higher than 2%, with the highest frequencies in 1KG-AMR-like populations (fig. 5, supplementary fig. S5, Supplementary Material online). The CYP2J2 enzyme accounts for roughly 1–2% of hepatic CYP protein expression and is involved in the oxidation pathways of polyunsaturated fatty acids (Murray 2016) and mediates the oxidation of drugs including ebastine (Hashizume et al. 2002; Lee et al. 2012), astemizole (Matsumoto and Yamazoe 2001; Lafite et al. 2007), and terfenadine (Matsumoto and Yamazoe 2001) and has high expression in the lung, kidney, heart, placenta, salivary gland, and skeletal muscle (Bièche et al. 2007; Murray 2016). Many *CYP2J2* polymorphisms occur at low frequencies, with the most common variant, CYP2J2*7, occurring at frequencies between 1% and 20% across global populations (King et al. 2002; Dreisbach et al. 2005; Wang et al. 2006; Polonikov et al. 2007, 2008; Murray 2016). A comparison of haplotypes demonstrates that a Neanderthal-like haplotype is found in 1KG-EUR-like and 1KG-AMR-like populations, with the highest frequency in the 1KG-AMR-like individuals (∼8%). Interestingly, the Neanderthal haplotype contains additional function-altering SNVs that are not found in modern humans (fig. 5, supplementary table S3, Supplementary Material online).

We also observed a pattern similar to archaic introgression in *CYP2C8* and *CYP2C9*, which have previously been identified as possible introgression candidates (Haeggström et al. 2022). The CYP2C9 enzyme is considered one of the most important DMEs in humans and responsible for metabolizing multiple classes of medications (Miners and Birkett 1998; Henderson et al. 2018) with substrates including nonsteroidal anti-inflammatories (Tracy et al. 1995; Miners et al. 1996; Hamman et al. 1997; Yamazaki et al. 1998), angiotensin II blockers (e.g., losartan) (Stearns et al. 1995), S-warfarin (Rettie et al. 1994; Yamazaki et al. 1998), phenytoin (Giancarlo et al. 2001), and tolbutamide (Miners and Birkett 1996). *CYP2C9* is located within 50 kb of *CYP2C8*, and their archaic SNVs show similar frequencies in modern populations (supplementary table S3, Supplementary Material online) and were thought to have linked archaic haplotypes (CYP2C9*2 and CYP2C8*3), so they may represent one complete archaic haplotype (Haeggström et al. 2022). However, none of these three genes were found to be candidates for introgression at a statistically significant level using the D+ statistic (Lopez Fang et al. 2022, supplementary table S7, Supplementary Material online). It should be noted that these genes are most certainly under ongoing selection across human populations (Thomas 2007), reducing the statistical power of tests designed to operate under neutrality. We also compared the archaic and modern haplotypes of the other *CYP450* genes (supplementary figs. S6 and S7, Supplementary Material online) but did not observe a similar pattern of introgression in any of them.

### Testing for Positive Selection in Archaic-like Haplotypes

We further tested whether the archaic-like haplotypes in *CYP2C8, CYP2C9*, and *CYP2J2* had a signature of positive selection by using the Population Branch Statistic (PBS). PBS measures genetic distances between three populations and identifies whether an SNV is found at an extremely high or low frequency in the target population compared to the other two populations: a closely related population to the target and an outgroup. We performed PBS for three genes: *CYP2J2* in 1KG-AMR-like, and *CYP2C8* and *CYP2C9* in 1KG-EUR-like. For *CYP2J2*, we selected Peruvians-like individuals (1KG-PEL-like) as our target population, as they present the highest frequency of archaic variants among the four 1KG-AMR-like populations, and likewise is the 1KG-AMR-like population with the largest component of Indigenous ancestry (Martin et al. 2017), and the lowest component of 1KG-AFR-like ancestry, as archaic variants are not found in 1KG-AFR-like populations. For *CYP2C8* and *CYP2C9*, we selected Central Europe-like individuals from 1KG (1KG-CEU-like) as the target population. For both comparisons, we used the 1KG-Han Chinese-like individuals (1KG-CHB-like) as the comparative population and 1KG-Yoruban-like individuals (1KG-YRI-like) as an outgroup. The mean PBS values of SNVs in *CYP2J2* indicate that allele frequencies are significantly higher in 1KG-PEL-like individuals, which suggests a signal of positive selection at this locus (table 1, supplementary fig. S8, Supplementary Material online). Additionally, when comparing the mean PBS score at *CYP2J2* relative to the mean PBS scores for 1,000 randomly sampled loci across the genome, we find the signal of positive selection to be statistically significant (*P* = 0.012, table 1). We did not observe similarly elevated frequencies in 1KG-CEU-like individuals for *CYP2C8* or *CYP2C9*.

**Table 1:** Mean PBS Values for *CYP2J2, CYP2C8,* and *CYP2C9*

Gene	Configuration	Individuals	Mean PBS	*P*-value
*CYP2J2*	PEL:CHB:YRI	All	0.044	0.208
*CYP2J2*	PEL:CHB:YRI	Introgressed	0.233	**0**.012*
*CYP2J2*	PEL:CHB:YRI	Nonintrogressed	0.074	0.106
*CYP2C8*	CEU:CHB:YRI	All	0.017	0.561
*CYP2C8*	CEU:CHB:YRI	Introgressed	0.037	0.368
*CYP2C8*	CEU:CHB:YRI	Nonintrogressed	0.033	0.391
*CYP2C9*	CEU:CHB:YRI	All	0.003	0.800
*CYP2C9*	CEU:CHB:YRI	Introgressed	0.057	0.259
*CYP2C9*	CEU:CHB:YRI	Nonintrogressed	0.011	0.700

Configurationsummarizes the populations used in each comparison. Individuals reflect subsampling to include all 1KG individuals, 1KG individuals who carry the introgressed allele, and 1KG individuals who do not carry the introgressed allele. Mean PBS value reflects an overrepresentation of the alleles at high frequency, and the *P*-value denotes statistical significance. Significant results are denoted with bold text and a star.

While a pattern of allele sharing between archaic humans and modern humans outside of Africa may represent archaic introgression, there are other explanations for this pattern. One is known as incomplete lineage sorting (ILS), in which a haplotype that existed in an ancestral population (e.g., the shared ancestor of humans and Neanderthals) is lost from one or more descendant populations (e.g., in Africans). Given properties such as the length of the gene and the recombination rate in the region, we calculated the likelihood that the patterns we observe in each of the pharmacogenes represent ILS. Our results suggest that the alternative splicing event in *CYP2A6*, and the shared African and Neanderthal divergent haplotypes in *CYP2B6* are very unlikely to represent ILS, although the haplotypes we observed in *CYP2J2, CYP2C8*, and *CYP2C9* were not statistically significant (table 2).

**Table 2:** A Summary of Archaic Variants Identified in Modern 1KG Human Populations for *CYP450* Genes

	Number of Archaic Variants			Number of SNVs Identified in Each Population				Probability of ILS
Gene	Total Identified	Identified in Modern Populations	EAS	EUR	AMR	SAS	PNG	*P*-value
*CYP1A2*	46	2	2^a^	2^a^	1^a^	1^a^	0	0.915
*CYP2A6*	69	10	0	6	6	6	3^a^	**0**.001*
*CYP2B6*	426	20	4	14	13	14	5^a^	**0**.019*
*CYP2C8*	165	15	15	15^a^	15^a^	15	0	0.094
*CYP2C9*	162	24	24	24^a^	24^a^	24^a^	1^a^	0.163
*CYP2C19*	332	65	52	59	3	58	5^a^	0.089
*CYP2D6*	44	3	0	0	0	0	3^a^	0.994
*CYP2E1*	84	1	0	0	0	0	1^a^	**0**.013*
*CYP2J2*	94	11	9	8^a^	9^a^	9	1^a^	0.269
*CYP3A4*	93	6	2	1	0	6	6^a^	<0.001*
*CYP3A5*	108	0	*N/A*	*N/A*	*N/A*	*N/A*	*N/A*	0.775

The number“Identified in Modern Populations” represents the number of SNVs in the gene shared between archaic and modern 1000 Genomes Project (1KG) humans, with a frequency of less than 1% in 1KG-AFR-like populations and a frequency greater than 1% in at least one individual in a non-1KG-AFR-like population. The number of SNVs found in each population is listed, along with the median allele frequencies for all SNVs identified in the populations in the region. “1KG-East Asians-like”(EAS), “1KG-Europeans-like”(EUR), “1KG- Americans-like” (AMR), and “1KG-South Asians-like” (SAS) refer to the populations from the 1KG dataset, while “SGD-Papuan-like” (PNG) refers to the Simons Genome Diversity (SGD) Project Papuan population. If any SNVs in a gene were present at a frequency of 10% or greater in a population in a given region, the gene is highlighted with a section gn (^a^). We also include the probability of modern and archaic humans inheriting a haplotype for these genes by incomplete lineage sorting (ILS, see supplementary table S6, Supplementary Material online). Significant results are denoted with bold text and a star.

## Discussion

### Neanderthal and Denisovan CYP450 Star Allele Diversity

We investigated a panel of 11 *CYP450* genes in archaic individuals which are responsible for 75% of all drug metabolizing reactions and represent the bulk of drug metabolizing enzymes (DME) for which therapeutic recommendations exist (Evans and Relling 1999). DMEs may be subject to evolutionary processes (e.g., mutation, drift, natural selection) because of their role as detoxifiers of xenobiotic substances, yet the insights from genetic variation in archaic genomes have not been characterized to date. Shared or introgressed variation from archaic ancestors can help us further understand clinical variation in modern human populations today and elucidate evolutionary processes driving CYP450 evolution.

The genetic diversity of the archaic *CYP450* genes in our panel varies greatly between loci, likely reflective of their diverse functional roles in the body. Polymorphism is maintained in some but not all genes, both within and between these two archaic sister species. Inherently, varying evolutionary trajectories for each *CYP450* gene is logical because each gene is responsible for metabolizing a different set of substrates, which will vary based on environment. It is especially interesting that Neanderthals are heterozygous for some pharmacogene alleles as Neanderthals are known for having very low genome-wide heterozygosity (Prüfer et al. 2014, 2017; Mafessoni et al. 2020) (particularly the Altai Neanderthal). Our observation of genetic polymorphism could indicate that selection has maintained haplotype diversity in some of these genes. The genes that were predicted to have a poor/slow (*CYP2A6*, *CYP2C19*) or ultra-rapid (*CYP2B6*, *CYP2D6*) metabolizer status have also been known to have extreme variation in human populations (Zhou et al. 2017) and may indicate instances of natural population variation or adaptation to the environment.

We observe multiple cases where archaic and modern humans show evidence for adapting to the same environment using different genetic variants. For example, the presence of slow-metabolizing *CYP2C19* variants in Neanderthals and modern humans from the East and South Asia regions suggests that there may be an environmental factor driving the shift to this phenotype. Another example is the CYP2A6*9 star allele, which is observed in Denisovans and Asian populations but with different haplotypes. Conversely, cases like the gene duplication of *CYP2D6* in Neanderthals suggest that archaic humans also adapted to unique environmental challenges. The presence of an ultra-rapid metabolizing allele in both the Altai and Vindija Neanderthals when *CYP2D6* alleles with a slower metabolic rate are much more common in humans today may suggest adaptation to an environment that is not shared by modern Eurasians, possibly because the particular environmental challenge faced by these Paleolithic populations no longer exists.

However, it is important to note that the predicted phenotypes are based on modern human phenotypes, and it is unclear how the archaic-specific variation (particularly from the novel function-altering variants) would affect metabolism in archaic individuals. These variants of uncertain significance would benefit from further functional tests, individually as well as in the haplotypes found in archaic individuals, to measure both enzyme activity and protein abundance. Further examination of pharmacogene variation in living individuals would be useful to test hypotheses for why these metabolic variants may have been adaptive in past archaic or modern human populations. For example, a comparison of *CYP2D6* haplotypes in individuals with hunter–gatherer and agricultural lifestyles suggests that slower metabolic variants became more adaptive as human populations transitioned to food production (Fuselli et al. 2010).

### Evidence for Archaic Introgression into Modern Populations

The distribution of archaic variants in the 11 *CYP450* genes we studied in modern populations is largely consistent with our general understanding of archaic introgression in human populations. For example, the majority of shared *CYP450* variants are shared with Neanderthals, which is consistent with most populations having much more Neanderthal ancestry than Denisovan ancestry, with the exception of some individuals from Melanesia and the SGD-Philippines population (Sankararaman et al. 2016; Larena et al. 2021). An individual from the CEPH-Human Genome Diversity Panel-Papuans had much more Denisovan ancestry than other individuals from world populations (Meyer et al. 2012) and may have had gene flow from a different Denisovan population than the one encountered by the ancestors of Eurasians (Vernot et al. 2016), and thus, it is not surprising that they have a unique set of archaic alleles for the *CYP450* genes, many of which are Denisovan in origin.

Given that pharmacogenes are important for interactions with new environments (Fuselli et al. 2010; Fuselli 2019), identifying pharmacogenomic regions with potential archaic introgression (shared variation between archaic and modern humans) would assist in future studies looking at selection and gene flow. For example, we identify multiple SNVs within *CYP2C9* that are shared between non-1KG-AFR-like African populations and Neanderthals, and similar to previous studies, identified evidence of archaic ancestry in the region (Skov et al. 2018). Interestingly, *rs1799853*, the causative SNV for the decreased enzyme activity CYP2C9*2 haplotype, is found in the Altai and Chagyrskaya Neanderthals and in 1KG-EUR-like populations at frequencies of 8–15%, and in 1KG-AMR-like populations with high European ancestry (Martin et al. 2017). In modern humans, the CYP2C9*2 haplotype has decreased enzyme activity compared to the reference haplotype (Van Booven et al. 2010), and negatively affects warfarin metabolism (Rettie et al. 1994), increases the risk of hypoglycemia from sulfonylurea treatments (Chen et al. 2020), and increases the risk of overdose on phenytoin (Ninomiya et al. 2000). The CYP2C9 variants are especially interesting because they have a global distribution inconsistent with the serial bottlenecks human populations experienced as they expanded out of Africa and into Eurasia, Oceania, and the Americas, suggesting that selection may play a role in how the allele frequencies are distributed today (Sistonen et al. 2009). If the CYP2C9*2 variant was introgressed from Neanderthals, the elevated frequency in 1KG-EUR-like populations might suggest adaptation to Western Eurasian environments, although our PBS test did not detect a significant signature of positive selection and we cannot rule out that the elevated frequency of the Neanderthal allele in 1KG-EUR-like group was the result of a strong founder effect. In addition, the length and recombination rate of the CYP2C9*2 haplotype are such that we cannot exclude ancestral sharing of this variant by ILS (*P* = 0.089, supplementary table S6, Supplementary Material online). However, the adjacent *CYP2C9* and *CYP2C8* genes are clustered within 50 kB and their archaic SNVs show similar frequencies in modern 1KG populations (supplementary table S3, Supplementary Material online), so linkage between *CYP450* genes may influence the direction of natural selection. Another study using a large database found that the combined haplotype length of CYP2C9*2 and its linked allele, CYP2C8*3, are likely archaic in origin (Haeggström et al. 2022). Further exploration of this haplotype is needed to determine whether this haplotype was introgressed and then targeted by positive selection.

Then, we identify an introgressed haplotype of *CYP2J2* that is found in all non-1KG-AFR-like populations (fig. 4). A comparison of haplotypes demonstrates that a Neanderthal-like haplotype is found in 1KG-EUR-like and 1KG-AMR-like populations, with the highest frequency in the 1KG-AMR-like individuals (∼8%). Interestingly, the Neanderthal haplotype contains additional function-altering SNVs that are not found in modern humans. Two observations provide a possible explanation: first, the Neanderthal populations which admixed with modern humans are phylogenetically distinct from the populations containing the Altai, Chagyrskaya, and Vindija individuals (Prüfer et al. 2014, 2017; Mafessoni et al. 2020). Second, the three Neanderthal individuals sequenced here are notorious for their elevated inbreeding coefficient, and it is predicted that purifying selection would be weaker in these populations. Thus, the deleterious variants we observed in the Altai, Chagyrskaya, and Vindija may not be fixed in all Neanderthals, and the population that admixed with modern humans did not carry these mutations, or they were carried in polymorphism alongside the wild type variants. This is consistent with evidence at other loci where Neanderthal ancestry in modern humans is more diverse than observed in the three late Neanderthal individuals for which we have high-quality genomes (Taskent et al. 2020; Villanea et al. 2021).

Purifying selection has already removed much of the archaic ancestry in the modern human gene pool (Green et al. 2010; Meyer et al. 2012; Sankararaman et al. 2012; Prüfer et al. 2017). However, archaic humans had smaller population sizes and this allowed for the accumulation of weakly deleterious alleles (Castellano et al. 2014; Vernot and Akey 2014; Sankararaman et al. 2014, 2016; Vernot et al. 2016). After archaic and modern human admixture occurred, increased pressure from purifying selection would have removed archaic deleterious haplotypes, and it has been suggested that a significant portion of Neanderthal ancestry was removed from the modern human gene pool in just a few generations (Harris and Nielsen 2016; Juric et al. 2016; Petr et al. 2019). The Neanderthal *CYP2J2* haplotype we observe in modern humans likely carries SNVs that represent neutral variants that were maintained in the population, or advantageous variants that may have been under positive selection. However, we cannot exclude ancestral sharing of this variant by ILS (*P* = 0.269, supplementary table S6, Supplementary Material online). Further exploration of these archaic *CYP450* variants can help clarify the interplay between purifying selection removing deleterious mutations and positive selection retaining advantageous archaic *CYP450* alleles.

Finally, we identify a structural variant in *CYP2A6* that may have originated uniquely in Neanderthals and was then introduced to modern humans through gene flow. In this study, we identify the CYP2A6*12 hybrid allele in all three Neanderthal individuals and find Neanderthal SNVs at this locus in modern 1KG humans from Europe, Southeast Asia, and the Americas regions, at frequencies between 1–3% (supplementary table S3, Supplementary Material online), but not in East Asia or Africa regions. This sharing of alleles, in particular the absence in 1KG-AFR-like, suggests that the human CYP2A6*12 may have been inherited through human-Neanderthal introgression. There are alternate explanations that do not include archaic gene flow, such as incomplete lineage sorting (ILS) or parallel evolution. Given the length of this haplotype (31.9 kb) and the regional recombination rate (0.77 cM/Mb; Myers et al. 2005); however, it is improbable that the 31.9 kb CYP2A6*12 haplotype would have been maintained by ILS in both lineages (*P* = 0.0014, table 2, supplementary table S6, Supplementary Material online). The final alternative to explain the prevalence of CYP2A6*12 is that this hybrid allele evolved multiple times, independently in modern non-Africans and Neanderthals. It has been proposed that the slow metabolizing of some plant secondary metabolites may be adaptive as high levels of these toxins in human tissues may act as a deterrent to parasites (Sullivan et al. 2008; Hagen et al. 2009, 2013), perhaps explaining why this hybrid allele may have evolved multiple times. Yet, the frequencies between 1% and 3% of CYP2A6*12 in modern non-1KG-AFR-like populations is consistent with genome-wide levels of Neanderthal introgression in global populations, making this the most likely alternative.

Not all pharmacogenetic variants with signals of selection in non-1KG-AFR-like are archaic in origin. It was previously observed that CYP3A4*1B and CYP3A5*3, which both code for reduced enzyme metabolism, are found at much higher frequencies in the Coriell Human Variation Collection (CHVC) European-descent-like and CHVC Han Chinese-like populations compared to the CHVC African-American-like population (Thompson et al. 2004). It was suggested that *CYP3A* genes’ role in sodium homeostasis may influence the selection of the rarer star alleles as populations adapted to nonequatorial environments. However, the SNVs responsible for both of these slow metabolizing alleles are found today at high frequencies in 1KG-AFR-like populations (18% for *rs776746* in CYP3A5*3, and 23% for *rs2740574* in CYP3A4*1B), and all the archaic humans likely have a phenotype of normal metabolism (although Denisovans have a single copy of CYP3A5*3), making it more likely that these alleles were already present in human populations as they expanded out of Africa. The slow-metabolizing alleles either rose in frequency due to a strong bottleneck effect during the African diaspora or were driven to high frequencies due to positive selection.

### Potential Super-archaic Introgression

For *CYP2B6*, we identified highly divergent haplotypes in the Vindija Neanderthal and a small number of 1KG-AFR-like individuals (*n* = 11). This duplicated haplotype is highly divergent from other archaic and modern human *CYP2B6* haplotypes, but the duplicated Neanderthal and human haplotypes are also distinct from one another (supplementary fig. 4, Supplementary Material online). The cause of the elevated SNV count in this duplicated haplotype is unclear. It is possible that the gene duplicate is nonfunctional, and the variation accumulated because selection has been relaxed, although there is no obvious evidence (i.e., a stop codon) to suggest that the enzyme's function has been impaired. This gene duplication might also be an ancestral variant that has survived at low frequencies in modern human and Neanderthal populations, but this is unlikely given our ILS calculations for *CYP2B6* (*P* = 0.02, see supplementary table S6, Supplementary Material online). Finally, it is also possible that the duplicated haplotype is introgressed from an archaic hominin that is more divergent than Neanderthals and Denisovans, also known as a “super-archaic” human. There is evidence to suggest that Denisovans (Prüfer et al. 2017) and Africans (Durvasula and Sankararaman 2020) have small percentages of their genomes that were inherited from super-archaic humans, and *CYP2B6* was previously identified as a candidate region for super-archaic introgression in African individuals (Durvasula and Sankararaman 2020). Gene flow has also been inferred between Neanderthals and the ancestors of modern humans before either expanded outside of Africa (Kuhlwilm et al. 2016; Hubisz et al. 2020; Petr et al. 2020). This implies that this gene duplication may have super-archaic origins. Future work on this haplotype's function and evolutionary history may clarify its origin, and how it came to exist in both Neanderthals and modern humans.

### Limitations

Our findings suggest that Neanderthals and Denisovans have pharmacogene diplotypes that are similar to that of modern humans, and in some cases may reflect gene flow between archaic and modern humans. However, some limitations to the data must be noted. First, the archaic genomes are ancient DNA, which is degraded compared to modern DNA. This degradation often manifests as lower read depth, skewed allele balance, shorter read lengths, as well as transition point mutations (cytosine [C] → thymine [T]), which impact both mapping and sequencing accuracy. To distinguish true polymorphism at heterozygous sites from sequencing errors and DNA damage, we excluded base pairs that are represented in only a single read for each position in the BAM read alignment and removed any heterozygous calls that had an allelic read ratio less than 0.2 or greater than 0.8. The nature of ancient genomes resulted in low read depth for some star alleles that were called, but given this limitation, we also manually checked the sequencing reads for all diplotypes reported.

Additionally, haplotype calling on the archaic genomes required phasing of the archaic *CYP450* sequences using a human reference panel, and diplotype calls were made using current known human star alleles. While this is the best reference panel available, it is possible that phasing an archaic genome using modern human reference genomes would result in an inaccurate haplotype. However, the majority of SNVs were homozygous, which is consistent with the much lower heterozygosity of Neanderthals and Denisovans along the entire genome (Meyer et al. 2012; Prüfer et al. 2014, 2017; Mafessoni et al. 2020), and many of the heterozygous star allele calls were composed of only a single nucleotide variant, effectively deeming the phasing nonimpactful. The concern that the archaic diplotypes may represent novel star alleles that are not shared with humans remains, and further functional validation of the novel potentially function-altering SNVs will be required to confirm the archaic diplotype calls.

We also used a novel read filtering method, resulting in a larger number of heterozygous sites compared to the variant call files that are usually used to represent the archaic genomes (Meyer et al. 2012; Prüfer et al. 2014, 2017; Mafessoni et al. 2020). The method used to make the previously generated variant call files likely used a more stringent method of filtering, resulting in more sites being called as homozygous. To ensure that our results were not biased by our variant calling method, we repeated all analyses (including calling star alleles, identifying archaic SNVs in modern humans, and comparing human and archaic haplotypes) using the original variant call files, and found that while the number of identified SNVs is smaller, the patterns identified (such as the divergent *CYP2B6* haplotype in the Vindija and allele sharing between archaic and modern humans) remain.

## Conclusions

Understanding the impact of archaic variation on modern human health is still in its early stages. While a few genes have been identified for which the archaic alleles play an important role in human adaptation to their environment, such as *EPAS1*, the vast contribution of archaic ancestry to human fitness is less clear. For example, modern humans carry archaic variants of medically important genes such as ABO blood group antigens, but their impact on human health remains unknown (Villanea et al. 2021). Our results suggest that interactions between modern and archaic humans resulted in the introduction of at least one novel *CYP450* variant into the modern human gene pool (CYP2A6*12), as well as the possible introduction or reintroduction of *CYP2C9* and *CYP2J2* variants that are now found at high frequencies in select human populations, helping them adapt to new environments as modern humans expanded out of Africa. Important insights will continue to emerge from careful inspection of pharmacologically relevant and highly studied genes such as the *CYP450* genes investigated here. In the future, characterizing more of the environmental influences on CYP450 evolution as well as identifying archaic *CYP450* alleles inherited by modern humans that have been adaptive in historically under-represented populations in genomic research such as *CYP2J2* in Indigenous American populations, which have uniquely adapted to numerous novel environments and have undergone multiple genetic bottlenecks due to colonization and disease, would be beneficial.

## Materials and Methods

### Samples

We investigate population-specific *CYP450* gene variation by combining data from the 1KG (1000 Genomes Project Consortium 2015), Papuans from the Simons Genome Diversity Project (Mallick et al. 2016), the complete Neanderthal genomes (Prüfer et al. 2014, 2017; Mafessoni et al. 2020), and the Denisovan genome (Meyer et al. 2012). We extracted coding variation from four archaic human genomes: the Denisovan individual from the Denisova cave in the Altai Mountains (∼21 × coverage), a Neanderthal individual from Croatia (∼42 × coverage), and two Neanderthal individuals from the Altai Mountains: one from the Denisova cave (∼30 × coverage), and one from the Chagyrskaya cave (∼28 × coverage). These individuals are estimated to be at least 50,000 years old. We additionally utilized a set of seventy published chimpanzee genomes (Prado-Martinez et al. 2013), which were used as a proxy for variation that is ancestral to modern and archaic humans.

### Variant Calling

Sequencing data for the three Neanderthal individuals and one Denisovan individual are publicly available with alignment and processing of the BAM files previously outlined (Meyer et al. 2012; Prüfer et al. 2014, 2017; Mafessoni et al. 2020). The BAM files were used to calculate depth of coverage in the sample's genomic regions (see section *Star Allele Calling*), and then processed for variant calling. *De novo* variant calling was performed from BAM files instead of using the previously generated variant call files to standardize the filtering and stringency of calls across genomes, as the variant-calling method utilized for the archaic genomes was far more conservative than standard methods used to call SNVs for modern human genomes.

Sample BAM files were separated by chromosome for easier downstream processing using samtools (version 1.10 with hts lib 1.10) “view” function (Li et al. 2009). Variant calling was performed for each *CYP450* gene of interest, using PyPGx (version 0.1.37) “bam2vcf” which implements the Genome Analysis Toolkit (GATK, version 4.1.9.0) “HaplotypeCaller” function (options -emit-ref-confidence GVCF; -minimum-mapping-quality 10) followed by GATK “GenotypeGVCF” to merge individual samples (Poplin et al. 2017). Variants were called against the HumanG1Kv37 hg19 reference assembly. GATK “VariantFiltration” was additionally utilized to annotate variants with a quality score (QUAL) of 50. Variants from modern human individuals from the 1KG (1000 Genomes Project Consortium 2015) included for further analysis were called using the same procedure.

For individual SNV analysis, variant call format (VCF) files were further filtered by the phred-scaled QUAL >40 and gene regions were determined as the RefSeq start and end coordinates of the gene with 2,000 base pairs upstream to account for the promoter region using bcftools (version 1.10.2 with hts lib 1.10.2) (Li et al. 2009; O’Leary et al. 2016). Variant read depth was calculated with the PyPGx (version 0.14.0, https://pypgx.readthedocs.io/en/latest/readme.html) pipeline (described below). Variants were additionally filtered for polymorphic sites that had a BAM read depth ≤ 1 for one allele or a ratio of minor allelic read depth to major allelic read depth < 0.2.

Variant annotation was conducted through ANNOVAR (-protocol refGeneWithVer, knownGene, avsnp150, ljb26_pp2hvar) which utilizes dbSNP150, RefSeq, and the UCSC genome browser to identify and characterize known variants; the occurrence of novel SNVs were additionally confirmed with Gnomad (v2.1.1) (Sherry et al. 2001; Kent et al. 2002; Wang et al. 2010; O’Leary et al. 2016; Karczewski et al. 2021). Variant locations were identified with ANNOVAR, which annotates gene location based on RefSeq and UCSC genome browser data. Variants are defined as exonic, splicing, ncRNA, 3′ and 5′UTR, intronic, or in the promoter region. If the variant type differed between the RefSeq and UCSC gene annotation, the called location is determined by an ordering precedence: exonic/splicing > ncRNA > 3′/5′UTR > intronic > promoter region.

Potentially function-altering variants were identified using three algorithms to rank variant deleteriousness: Combined Annotation Dependent Depletion (CADD) integrates multiple weighted metrics to identify deleterious variants (Rentzsch et al. 2019, 2021), Sorting Intolerant From Tolerant (SIFT) predicts the functional impact of amino acid substitution caused by genetic variation (Vaser et al. 2016), and Polymorphism Phenotyping (PolyPhen) identifies the predicted structural and functional outcome of amino acid substitution (Adzhubei et al. 2010). Variants considered potentially function-altering (referred to as “damaging” or “pathogenic” by algorithms) were defined as SNVs that are in the top 1% of deleterious variants by CADD score (Phred-normalized CADD score ≥ 20), deleterious by SIFT prediction (SIFT score ≤ 0.05) or damaging by Polyphen2 prediction (Polyphen2 score between 0.15–1.0 as possibly damaging and 0.85–1.0 as probably damaging).

To confirm whether the SNVs we observe had been previously identified in archaic ancestry tracts, we compared the list of SNVs to the archaic ancestry tracts identified by Skov et al. (2018). These ancestry tracts were identified using a Hidden Markov Model-based method and individual tract data is available for each non-1KG-AFR-like individual in the 1000 Genomes Phase 3 population dataset. We looked at all individuals with the SNVs also found in archaic humans, and calculated the percentage of alleles that were within a previously-identified archaic ancestry tract. All allele frequencies and data are reflective of genetic similarity, or a quantitative measure of the genetic resemblance between individuals that reflects the extent of shared genetic ancestry.

### Star Allele Calling

*CYP450* “star alleles” (phased haplotypes) were called using Stargazer (Lee, Wheeler, Patterson, et al. 2019; Lee, Wheeler, Thummel, et al. 2019) and PyPGx (version 0.14.0), bioinformatics tools for identifying star alleles by detecting single nucleotide variants (SNVs), insertion/deletions (indels), and structural variants (SVs) of pharmacogenes. Phased haplotypes were called for eleven pharmacogenes that represent enzymes which are responsible for up to 75% of the metabolism of commonly prescribed drugs (Evans and Relling 1999): *CYP1A2*, *CYP2A6*, *CYP2B6*, *CYP2C19*, *CYP2C8*, *CYP2C9*, *CYP2D6*, *CYP2E1*, *CYP2J2*, *CYP3A4*, and *CYP3A5.* VCF files, without additional QC filtering, along with the read depth data for each gene were the input into the haplotype callers, which utilize the statistical phasing software BEAGLE to phase the variants into haplotypes (Browning and Browning 2007). Star alleles were then inferred by identifying the haplotype calls from core variants of each star allele, as well as tag variants, which define sub-alleles of each star allele. To avoid incorporating sequencing error into our star allele calls, diplotype calls were considered “nondeterminant” if any variant used in star allele detection method had a read depth ≤ 2. This is because sequencing error is random for position and identity, and as such, any given genomic position is not expected to carry multiple errors of the same identity, as opposed to true heterozygous positions which will carry multiple instances of two base-pair identities.

SV calls were assessed with PyPGx (version 0.14.0) using three control genes: vitamin D receptor (*VDR)*, epidermal growth factor receptor (*EGFR*), and ryanodine receptor 1 (*RYR1*). Depth of coverage data was calculated using PyPGx to call the read depth at each position and was compared to the control genes, which are stable read regions that are highly conserved and do not fluctuate in copy number, to assess copy number variation in the eleven pharmacogenes (MacDonald et al. 2014). Final calls for star alleles were determined as the call that was consistent in the highest number of control genes. Phenotype prediction was conducted in PyPGx by converting the called diplotype into an activity score, which is then used to predict the gene phenotype (Lee, Wheeler, Patterson, et al. 2019; Gaedigk et al. 2018). Calls were additionally confirmed with visual inspection of read depth regions for the three control genes.

### Identifying Archaic Alleles in Modern Populations

To identify the presence of archaic *CYP450* variants in modern human populations, we calculated the allele frequency of the archaic variants described above in modern humans. We used modern human genomes from all populations sequenced for the 1KG (1000 Genomes Project Consortium 2015), as well as the Papuans sequenced as part of the Simons Genome Diversity Project (Mallick et al. 2016). We also focused on a subset of SNVs that were more likely to be introgressed: those SNVs found in other 1KG populations but not in 1KG-AFR-like populations. These SNVs had to have a frequency in 1KG-AFR-like populations of less than 1% and be present in at least one non-1KG-AFR-like population with an allele frequency greater than 1%. For all results described in the text, we used the archaic VCF generated using the methods in this paper for comparison with modern humans. For comparison, we repeated these analyses with the published VCFs that are associated with the original archaic genome sequencing studies (Meyer et al. 2012; Prüfer et al. 2017). Alleles were categorized as “derived” or “ancestral” based on the ancestral allele calls identified by the 1KG (1000 Genomes Project Consortium 2015). We additionally examined these pharmacogenes in a cohort of seventy publicly available chimpanzee genomes aligned to GRCh37—hg19 human genome assembly (Prado-Martinez et al. 2013) to determine if any of the variants shared between archaic and modern humans might be found in chimpanzees as well, representing shared ancestral variation.

We used Haplostrips (Marnetto and Huerta-Sánchez 2017) to calculate the distance between haplotypes of all *CYP450* genes in modern humans in the 1KG Panel relative to a reference haplotype. Haplotypes are reordered by decreasing similarity with the archaic reference haplotype. In this case, the Haplostrip is polarized to a Vindija Neanderthal reference haplotype (a consensus of the two archaic chromosomes) or a Denisovan reference haplotype, and each subsequent haplotype is ordered by genetic similarity, from most related to least related. For this analysis, we looked at haplotypes of each *CYP450* gene at the gene coordinates, plus 5,000 base pairs downstream and upstream to capture more linked neutral variation, and compared the 1KGs individual's haplotypes with archaic haplotypes.

For one pharmacogene, *CYP2B6*, we observed highly divergent haplotypes found in the Vindija Neanderthal and a small number of 1KG-AFR-like individuals (*n* = 11). To determine how the divergent Vindija *CYP2B6* haplotype compared to the divergence between the Vindija Neanderthal and other Neanderthals in other regions of the genome, we calculated the pairwise distance between the Chagyrskaya Neanderthal and Vindija Neanderthal genomes with 29.1 kb windows (*CYP2B6* gene length) across the genome. To explore SNV sharing between the *CYP2B6* haplotypes, we divided the archaic and modern humans into four groups: the Vindija genome (containing the divergent haplotype), the other three archaic genomes, the eleven 1KG-AFR-like with the divergent haplotypes, and the rest of the modern 1KG-AFR-like without the divergent haplotypes. Each group was scored for the presence or absence of a given SNV, and the sharing of these SNVs was summarized in a Venn Diagram (supplementary fig. S3, Supplementary Material online).

In addition, we performed the D+ statistic (Lopez Fang et al. 2022) as a test of local introgression for three genes: *CYP2C8*, *CYP2C9*, and *CYP2J2*. We calculated D+ for all combinations of P1 = {YRI}, P2 = {CEU, PEL}, and P3 = {Vindija Neanderthal} where ancestral states were polarized using the ancestral allele calls in the 1000 Genomes Panel. To assess significance, we calculated D+ values from the genomic background of windows with comparable effective sequence length to the total length of each gene. The genomic background distribution of D+ values were then used to construct a z-distribution, and subsequently calculated *P*-values to assess if the observed D+ value in the CYP450 region significantly differed from 0.

### Positive Selection in Archaic CYP450 Alleles in Modern Humans

To assess if any *CYP450* introgressed haplotypes found at high frequency have been targeted by positive selection we utilized the population branch statistic (PBS), which uses pairwise estimates of F_ST_ to measure the branch length in the target population since its divergence from two control populations (Yi et al. 2010). For *CYP2C8* and *CYP2C9* we chose 1KG-CEU-like individuals as the target population—as it harbors the greatest number of Neanderthal-like haplotypes—and 1KG-CHB-like individuals as our control population. For *CYP2J2* we chose 1KG-PEL-like individuals as the target population—as it harbors the greatest number of Neanderthal-like haplotypes—and 1KG-CHB-like individuals as our control population. For each target population we ran three separate PBS analyses: (1) with all individuals in the target population, (2) only for individuals who harbor at least one copy of the Neanderthal-like haplotype, and (3) only for individuals who harbor no copies of the Neanderthal-like haplotype. For analyses two and three we also randomly subsampled the two control populations to match the same number of individuals as our target population. We chose this experimental design to assess whether the signal of positive selection is being driven by the Neanderthal-like haplotype. For each set of analyses we calculated PBS values for every SNV in each *CYP450* region and the mean PBS values for that region. To determine the significance of the SNVs and the *CYP450* regions, we also calculated PBS values for every SNV in the genome, and the mean PBS value for 1,000 randomly sampled (with replacement) bootstrapped regions of comparable length. We used an outlier approach to identify SNVs that are under positive selection at each *CYP450* region by setting the significance threshold at the genome-wide 99th PBS percentile and we assessed if the mean PBS value for each *CYP450* region is statistically significant by determining how often the bootstrap distribution overlaps with the observed mean PBS value. For each of the three genes, the significance threshold was adjusted for multiple comparisons (three comparisons).

### ILS Calculation

While shared ancestry between archaic humans and modern humans outside of Africa may suggest that introgression has occurred, this pattern may also represent ILS. To attempt to distinguish between the two possibilities, we assessed the probability of ILS occurring in a given region using a previously published equation (Huerta-Sánchez et al. 2014):


$$L=\;\frac{1}{R\;\times \;\frac{b}{G}}.$$

Briefly, the expected length of a shared ancestral sequence haplotype (*L*) is calculated as the inverse of regional recombination rate (*R*) multiplied by the branch length (*b*) over the generation time (*G*) The probability (*p*) of seeing a shared haplotype of length (*m*) follows a Gamma distribution with a shape (*S*) of 2 and a rate (*r*) of 1/*L*:


$$p=1-pGamma\left(m,\;S=2\;,\;r=\;\frac{1}{L}\right)$$

Here, we determined the regional recombination rates (*R*) using HapMap (Myers et al. 2005), used a generation time (*G*) of 29 years (and a minimum and maximum values of 25 and 35 years in the supplement) (Langergraber et al. 2012; Zeberg and Pääbo 2021), and assumed a branch length (*b*) of 550,000 years with a 50,000 year timeframe of Neanderthal-human interbreeding (Prüfer et al. 2014; Zeberg and Pääbo 2021).

### Data Visualization

All data visualization was conducted in R Studio Suite (R version 4.0.2, https://www.r-project.org/) with the use of the CRAN library *ggplot2* for graphical visualization of variant frequency, star allele diplotype calls, deleterious variant occurrence, and haplotype distance visualization (Wickham (2016; R Core Team 2021). The R package *venneuler* was used to create the Venn diagram in supplementary figure 3, Supplementary Material online (https://CRAN.R-project.org/package=venneuler).

## Supplementary Material

evad222_Supplementary_Data

## Data Availability

The scripts created to analyze and generate these data can be found at https://github.com/ClawIndigenousGenomicsEthicsLab/Archaic_PGx. Genomes used in this study are from the 1000 Genomes Project and the Simons Genome Diversity Project, and the published complete Neanderthal and Denisovan Genomes, all of which are already publicly available.

## Supplementary Material

Supplementary tables S1 to S8 and figures S1 to S8 are available at *Genome Biology and Evolution* online (http://www.gbe.oxfordjournals.org/).
